# Supplementary material for: Genomic epidemiology of putative hypervirulent Klebsiella pneumoniae species complex in Dutch patients, January–December 2022
Source: Microbiol Spectr. 2026 Jan 12;14(2):e02259-25. doi: 10.1128/spectrum.02259-25 (PMC12889062; doi:10.1128/spectrum.02259-25)
Supplement: Supplemental figures — Fig. S1 to S6. [file spectrum.02259-25-s0001.pdf]

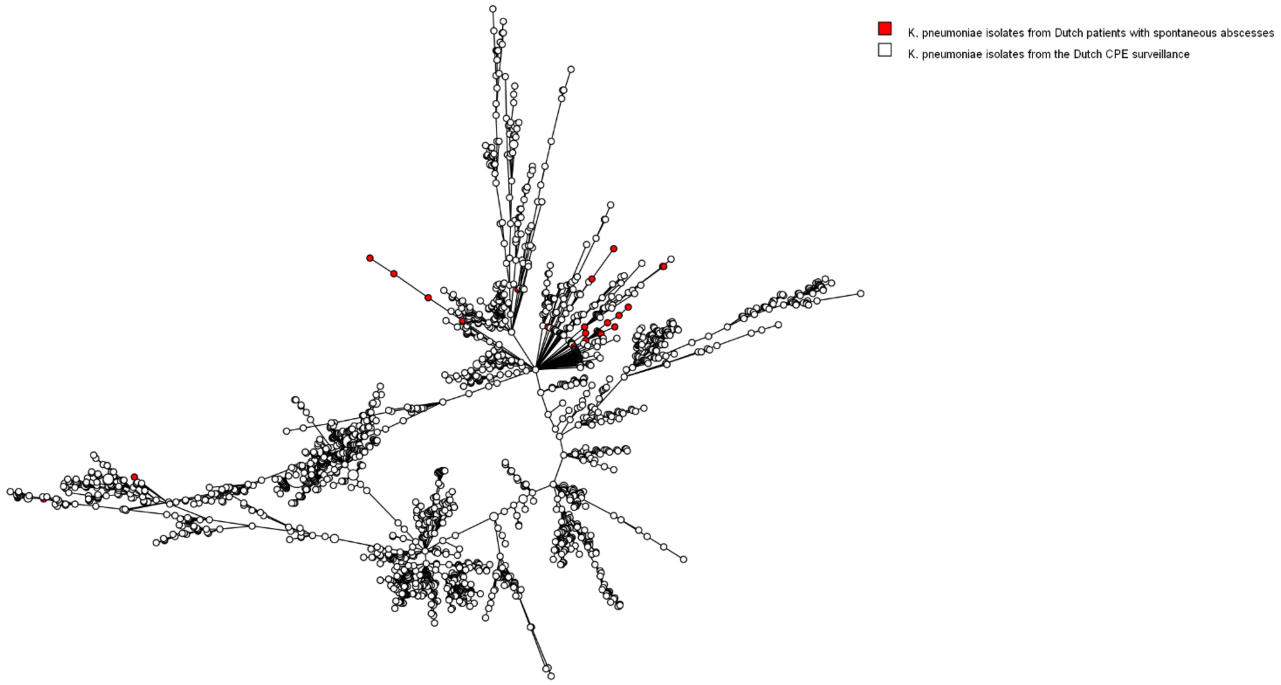

Figure S1. Minimum spanning tree based on whole genome multi-locus sequence typing (wgMLST) results of 28 *K. pneumoniae* isolates from patients with spontaneous abscesses and 1,701 *K. pneumoniae* isolates from the Dutch national carbapenemase-producing Enterobacterales (CPE) surveillance. The colors represent the isolate types. A genetic cluster was defined as two or more isolates with an allelic distance of 20 or less, but no genetic clusters were found between study isolates and CPE surveillance isolates.

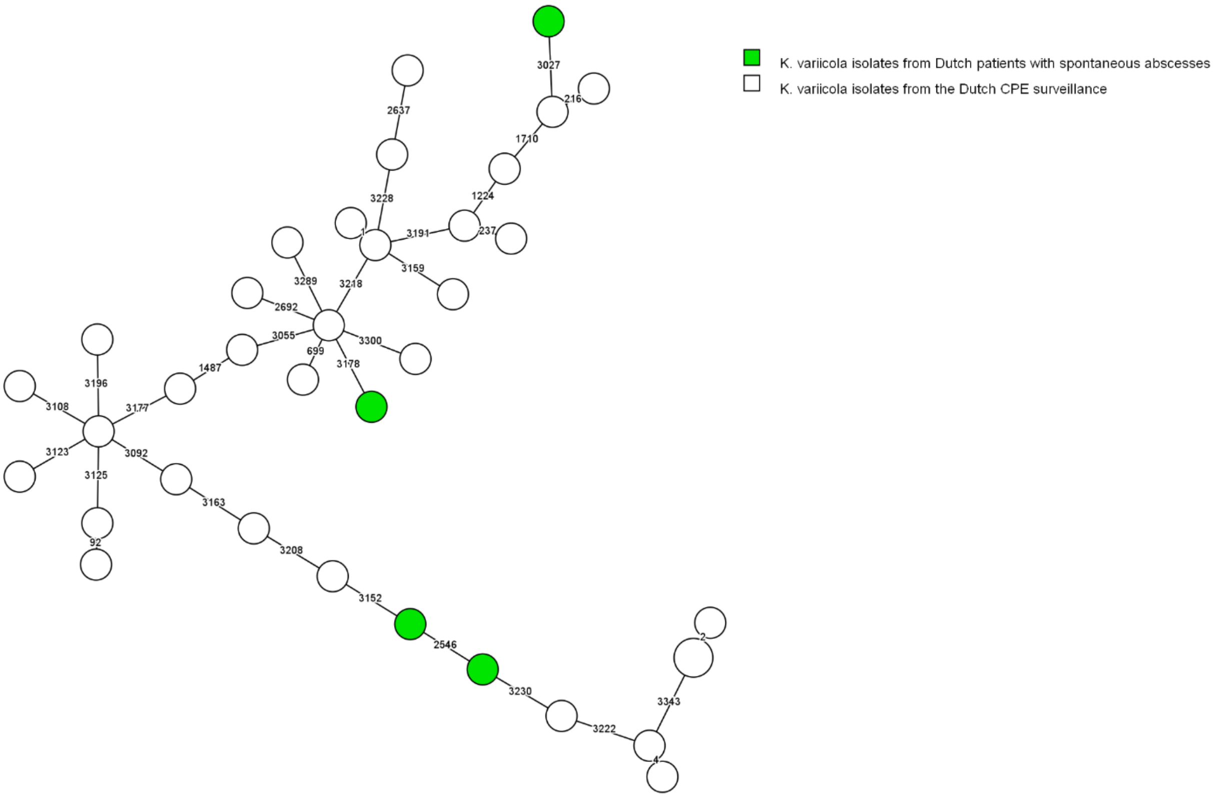

Figure S2. Minimum spanning tree based on whole genome multi-locus sequence typing (wgMLST) results of 4 *K. variicola* isolates from patients with spontaneous abscesses and 32 *K. variicola* isolates from the Dutch national carbapenemase-producing Enterobacterales (CPE) surveillance. The colors represent the isolate types. A genetic cluster was defined as two or more isolates with an allelic distance of 20 or less, but no genetic clusters were found between study isolates and CPE surveillance isolates.

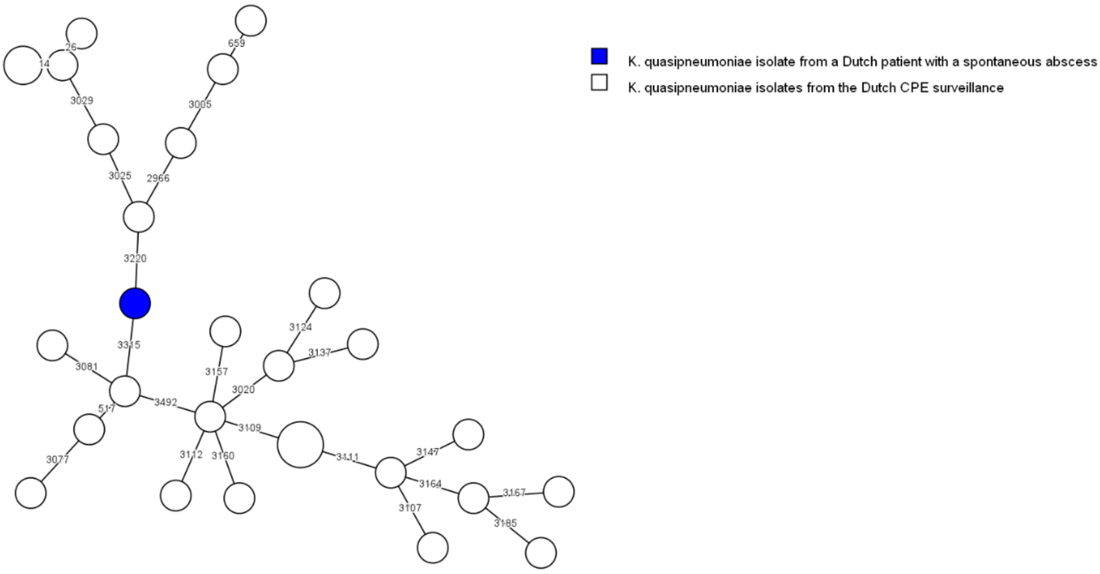

Figure S3. Minimum spanning tree based on whole genome multi-locus sequence typing (wgMLST) results of 1 *K. quasipneumoniae* isolate from a patient with a spontaneous abscess and 29 *K.*

***quasipneumoniae* isolates from the Dutch national carbapenemase-producing Enterobacterales (CPE) surveillance.** The colors represent the isolate types. A genetic cluster was defined as two or more isolates with an allelic distance of 20 or less, but no genetic clusters were found between study isolates and CPE surveillance isolates.

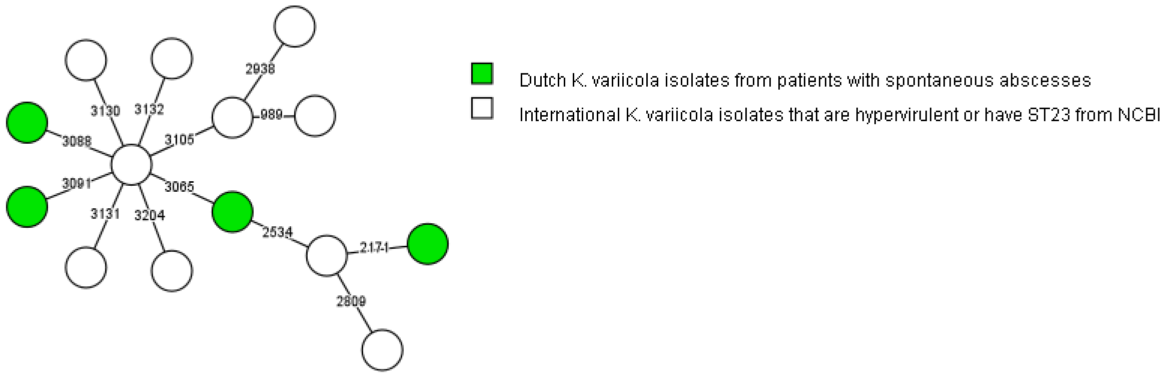

Figure S4. Minimum spanning tree based on whole genome multi-locus sequence typing (wgMLST) results of 4 *K. variicola* isolates from Dutch patients with spontaneous abscesses and 10 international *K. variicola* isolates that are hypervirulent and/or have ST23 from NCBI. The colors represent the isolate types. A genetic cluster was defined as two or more isolates with an allelic distance of 20 or less, but no genetic clusters were found including both Dutch and international isolates.

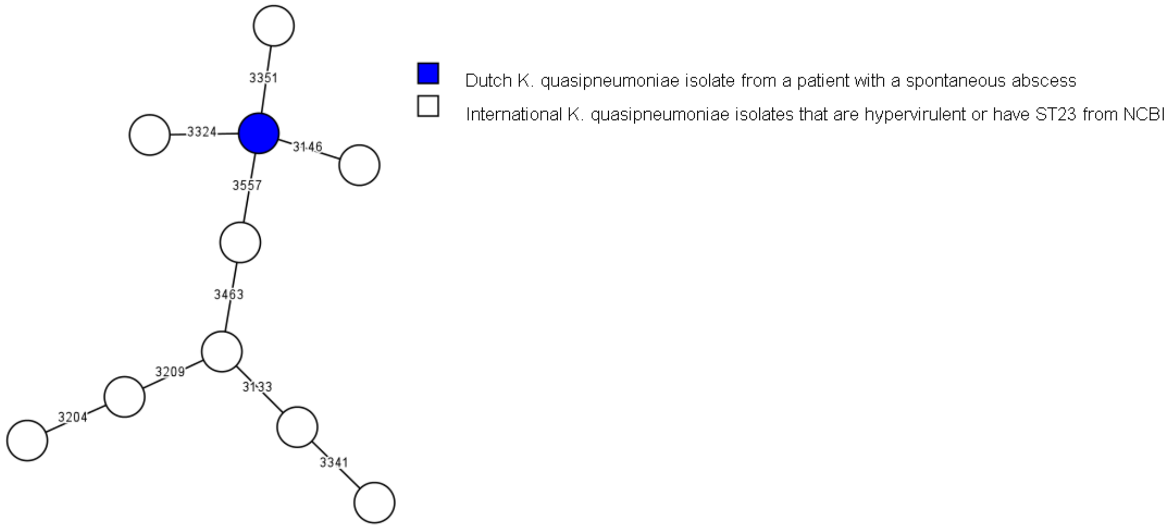

Figure S5. Minimum spanning tree based on whole genome multi-locus sequence typing (wgMLST) results of 1 *K. quasipneumoniae* isolate from a Dutch patient with a spontaneous abscess and 9 international *K. quasipneumoniae* isolates that are hypervirulent and/or have ST23 from NCBI. The colors represent the isolate types. A genetic cluster was defined as two or more isolates with an allelic distance of 20 or less, but no genetic clusters were found including both Dutch and international isolates.

A.

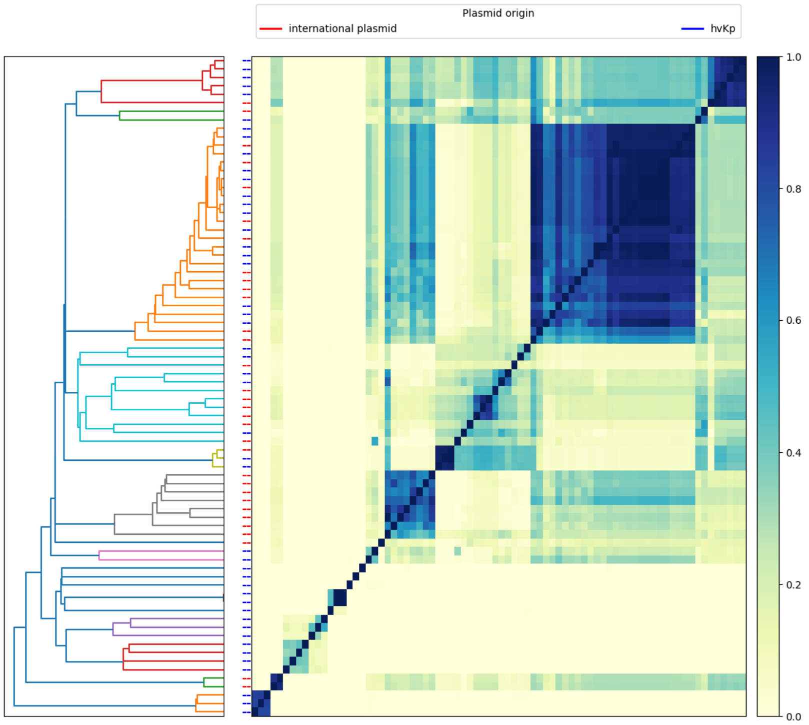

B.

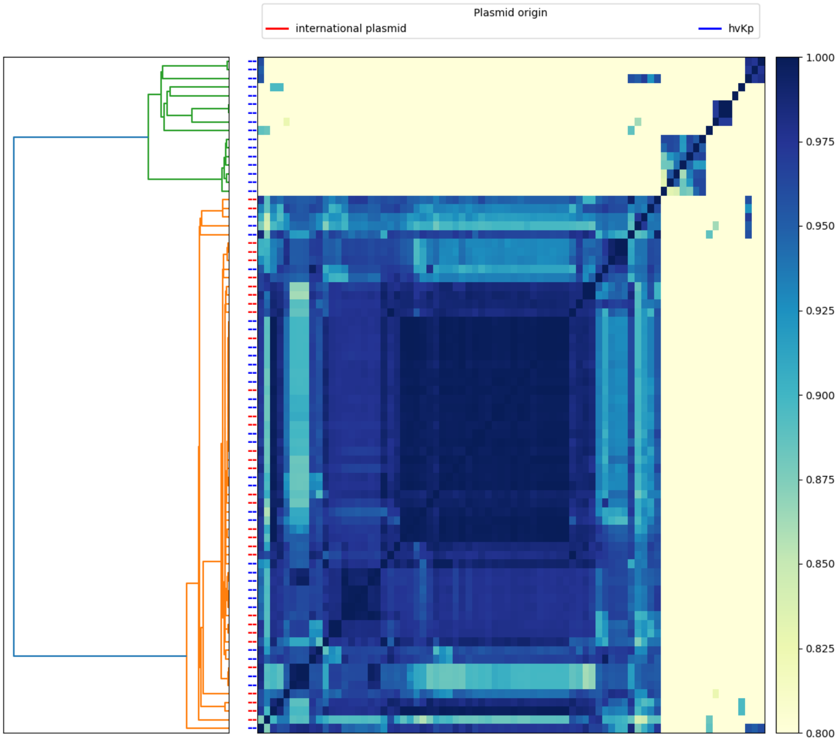

Figure S6. Relatedness of plasmids from *K. pneumoniae* species complex isolates from patients with spontaneous abscesses (hvKp) and international virulence plasmids from hvKp isolates (international plasmid). A. Percentage of coverage with a minimal identity of >80%. B. Percentage of identity. The plasmids, with different colors per isolate type (plasmid origin), represent the X- and Y-axis. The colors in the matrix represent the average nucleotide identity between the plasmids, determined using pyANI.
