# Supplementary material for: Genomic epidemiology of putative hypervirulent Klebsiella pneumoniae species complex in Dutch patients, January–December 2022
Source: Microbiol Spectr. 2026 Jan 12;14(2):e02259-25. doi: 10.1128/spectrum.02259-25 (PMC12889062; doi:10.1128/spectrum.02259-25)
Supplement: Supplemental material — Supplemental text. [file spectrum.02259-25-s0002.pdf]

Supplemental material and methods

Bacterial isolates

Only members of the *Klebsiella pneumoniae* species complex (KpSC) were included in this study: *Klebsiella pneumoniae* (Kp1), *Klebsiella quasipneumoniae subsp. quasipneumoniae* (Kp2), *Klebsiella quasipneumoniae subsp. similipneumoniae* (Kp4), *Klebsiella variicola subsp. variicola* (Kp3), *Klebsiella variicola subsp. tropica* (Kp5), *Klebsiella quasivariicola* (Kp6) and *Klebsiella africana* (Kp7).(1)

Genomic analyses

All isolates included in this study were subjected to paired-end next-generation sequencing (NGS), performed on the Illumina NextSeq550 platform (Illumina, USA) by the RIVM inhouse sequencing platform. Read quality analysis and de novo assembly was performed with Juno-assembly v2.0.2 pipeline. NGS results were imported into BioNumerics version 7.6.3 (Applied Maths, Sint-Martens-Latem, Belgium) for analyses.

For the assessment of virulence factors, sequences of virulence genes in *K. pneumoniae* of BIGSdb-Pasteur (2, 3) and the virulence factor database (4, 5) were used. In addition, an extensive literature search on virulence genes in *K. pneumoniae* was performed in February 2023 and gene sequences were searched for in the respective articles or in the NCBI or KEGG databases. All isolates were screened for these putative virulence genes using BLAST v2.13.0 (6) on the de-novo-assembly contigs and only virulence genes with > 90% sequence identity and >80% sequence coverage with the reference sequences were included. Furthermore, for tellurite and silver resistance genes (*terZBCDE and silS*), the AMRfinder software (7) was used. PointFinder (8) was used for detections of mutations in genes encoding outer membrane proteins OmpK35 and OmpK36, proteins that are potentially involved in virulence.(9) The examined 240 putative virulence genes/factors and three putative virulence-inhibiting genes (*FNR,*(10) *fur,*(10-12) and *sugE*,(13)) can be found in table S1. Kleborate v2.3.2 was used to assess the species, virulence scores, capsular serotypes (K-antigen) and LPS serotypes (O-antigen) and predictions on virulence factor truncation were used.(14) Two important molecular definitions of hvKp are Kleborate virulence score (12) and hvKp criteria recently proposed by Russo et al.(10) The Kleborate virulence score describes key hypervirulence loci and ranges from 0 to 5: 0 = no yersiniabactin, colibactin or aerobactin; 1 = yersiniabactin only; 2 = yersiniabactin and colibactin (or colibactin only); 3 = aerobactin without yersiniabactin or colibactin; 4 = aerobactin with yersiniabactin (no colibactin); 5 = yersiniabactin, colibactin and aerobactin.(14) Isolates were checked for the

hvKp criteria from Russo et al. (10) Isolates were screened for antimicrobial resistance genes with the ResFinder (15) software using a cut-off of ≥99% identity and ≥99% coverage. Detected beta-lactamase genes were checked for ESBL-association via the beta-lactamase database.(16)

For classical multi-locus sequence typing (MLST), the existing KpSC schemes available via SeqSphere were used. For whole-genome wgMLST, minimum spanning trees were contemplated using BioNumerics version 8.1 using an in-house *K. pneumoniae* wgMLST scheme.(17) *K. variicola* and *K. quasipneumoniae* isolates were only included in the wgMLST analysis if they had >90% detected core genes of *K. pneumoniae* of the wgMLST scheme (two *K. quasipneumoniae* isolates from the CPE surveillance were excluded from the analysis due to a percentage of detected core genes of 89.2 % and 89.9%). A genetic cluster was defined as two or more isolates with an allelic distance of ≤ 20.(18) Furthermore, the genomes of the isolates were compared to genomes of hypervirulent (also including other STs than ST23) and/or ST23 KpSC isolates from the National Center for Biotechnology Information (NCBI) database (table S6). Furthermore, the isolates were compared to KpSC isolates from the carbapenemase-producing Enterobacterales (CPE) surveillance of the National Institute for Public Health and the Environment(19) to examine genetic relatedness with this large collection of (mostly classical) KpSC isolates from the Netherlands. These 1,762 sequenced CPE isolates from the period February 2012 until October 2025 included only four K. pneumoniae isolates with maximum Kleborate virulence score 5.

PlasmidFinder software (20) was used to assess the presence of plasmid replicons using a 100% identity and coverage cut-off. Third-generation sequencing (TGS) via Nanopore long-read sequencing was performed to assess characteristics of plasmids. Hybrid assemblies (21) were performed by combining NGS and TGS data in Unicycler v0.5.0 enabling reconstruction of chromosomes and plasmids and assessing plasmid characteristics. Contigs were annotated by Bakta v1.6.1 (22) and loaded into BioNumerics for analyses. Further details of DNA isolation, TGS and hybrid assembly were described before.(23) Non-circular contigs and contigs of <2.5 kb were excluded. Plasmids were characterized via MOB-suite v3.1.8.(24, 25) A literature search was performed in August 2023 to search for previously detected virulence plasmids in hvKp isolates. Plasmids of isolates included in this study were compared to each other and to international virulence plasmids from the literature (table S2) using "chromosome comparison" in BioNumerics (full sequence based). The cut-off for genetically related plasmids was ≥90% identity. In case of uncertainties in the alignments, the alignment was checked by making the starting point of the alignments the same using CLC

Genomics Workbench version 24.0.2. Furthermore, we checked comparability of %GC-content within the clusters. In addition, a figure was contemplated visualizing relatedness of Dutch and international plasmids using Average Nucleotide Identity (determined using pyANI).(26)

Dutch plasmids were compared to previously found plasmids in Enterobacterales isolates (or in KpSC isolates only in case of an error for all Enterobacterales) using NCBI BLAST (megablast) on the 23rd of October 2024 with a cut-off of ≥90% identity and ≥90% query coverage. (6, 27)

Supplemental results

Plasmid analysis

Among the Dutch isolates, we found five genetic clusters of plasmids with ≥90% identity (2-7 plasmids per cluster, 15 plasmids in total). None of these 15 plasmids from clusters carried resistance genes. The largest cluster contained seven plasmids from seven study isolates (5 ST23 and 1 ST86 and 1 ST828). These plasmids were 220-230 kb, contained the *repB* replicon and were predicted to be non-mobilizable. These plasmids harbored no resistance genes and carried the salmochelin and aerobactin gene cluster, *rmpADC*, *rmpA2*, tellurite resistance genes, the silver resistance gene *silS*, *peg-344/pagO* and several less known putative virulence genes.(28) These plasmids resembled (≥90% ID) well-known virulence plasmids pLVPK(29), pK2044(30) (also called KpVP-1(1)) and pSGH10(31), another $bla_{\text{KPC-2}}$-encoding virulence plasmid pKP70-2(32) (although the $bla_{\text{KPC-2}}$ gene is absent in the plasmids from our study) and virulence plasmid pRJA166b.(33) None of the Dutch plasmids clustered with the well-known Kp52.145 pII virulence plasmid.

References

1. Wyres KL, Lam MMC, Holt KE. Population genomics of Klebsiella pneumoniae. Nat Rev Microbiol. 2020;18(6):344–59.
2. Jolley KA, Maiden MC. BIGSdb: Scalable analysis of bacterial genome variation at the population level. BMC Bioinformatics. 2010;11:595.
3. Institut Pasteur. Klebsiella locus/sequence definitions database. Available from: https://bigsdb.pasteur.fr/cgi-bin/bigsdb/bigsdb.pl?db=pubmlst_klebsiella_seqdef&page=downloadAlleles&tree=1 (accessed on the 4rd of July 2023).
4. Liu B, Zheng D, Zhou S, Chen L, Yang J. VFDB 2022: a general classification scheme for bacterial virulence factors. Nucleic Acids Res. 2022;50(D1):D912–d7.

5. NHC Key Laboratory of Systems Biology of Pathogens, National Institute of Pathogen Biology, CAMS&PUMC. 2004. Virulence factor database. Available from: https://www.mgc.ac.cn/cgi-bin/VFs/compvfs.cgi?Genus=Klebsiella (accessed on the 4rd of July 2023).
6. Altschul SF, Gish W, Miller W, Myers EW, Lipman DJ. Basic local alignment search tool. J Mol Biol. 1990;215(3):403–10.
7. Feldgarden M, Brover V, Gonzalez-Escalona N, Frye JG, Haendiges J, Haft DH, et al. AMRFinderPlus and the Reference Gene Catalog facilitate examination of the genomic links among antimicrobial resistance, stress response, and virulence. Sci Rep. 2021;11(1):12728.
8. Zankari E, Allesøe R, Joensen KG, Cavaco LM, Lund O, Aarestrup FM. PointFinder: a novel web tool for WGS-based detection of antimicrobial resistance associated with chromosomal point mutations in bacterial pathogens. J Antimicrob Chemother. 2017;72(10):2764–8.
9. Tsai YK, Fung CP, Lin JC, Chen JH, Chang FY, Chen TL, et al. Klebsiella pneumoniae outer membrane porins OmpK35 and OmpK36 play roles in both antimicrobial resistance and virulence. Antimicrob Agents Chemother. 2011;55(4):1485–93.
10. Zhu J, Wang T, Chen L, Du H. Virulence Factors in Hypervirulent Klebsiella pneumoniae. Front Microbiol. 2021;12:642484.
11. Holt KE, Wertheim H, Zadoks RN, Baker S, Whitehouse CA, Dance D, et al. Genomic analysis of diversity, population structure, virulence, and antimicrobial resistance in Klebsiella pneumoniae, an urgent threat to public health. Proc Natl Acad Sci U S A. 2015;112(27):E3574–81.
12. Russo TA, Marr CM. Hypervirulent Klebsiella pneumoniae. Clin Microbiol Rev. 2019;32(3).
13. Wu MC, Lin TL, Hsieh PF, Yang HC, Wang JT. Isolation of genes involved in biofilm formation of a Klebsiella pneumoniae strain causing pyogenic liver abscess. PLoS One. 2011;6(8):e23500.
14. Lam MMC, Wick RR, Watts SC, Cerdeira LT, Wyres KL, Holt KE. A genomic surveillance framework and genotyping tool for Klebsiella pneumoniae and its related species complex. Nat Commun. 2021;12(1):4188.
15. Bortolaia V, Kaas RS, Ruppe E, Roberts MC, Schwarz S, Cattoir V, et al. ResFinder 4.0 for predictions of phenotypes from genotypes. J Antimicrob Chemother. 2020;75(12):3491–500.
16. Naas, T.; Oueslati, S.; Bonnin, R. A.; Dabos, M. L.; Zavala, A.; Dortet, L.; Retailleau, P.; Iorga, B. I., Beta-Lactamase DataBase (BLDB) – Structure and Function. J. Enzyme Inhib. Med. Chem. 2017, 32, 917-919.
17. Hendrickx APA, Landman F, de Haan A, Witteveen S, van Santen-Verheuvel MG, Schouls LM, et al. $bla_{\text{OXA-48-like}}$ genome architecture among carbapenemase-producing *Escherichia coli* and *Klebsiella pneumoniae* in the Netherlands. Microb Genom. 2021;7(5).
18. Hendrickx APA, Landman F, de Haan A, Borst D, Witteveen S, van Santen-Verheuvel MG, et al. Plasmid diversity among genetically related *Klebsiella pneumoniae* $bla_{\text{KPC-2}}$ and $bla_{\text{KPC-3}}$ isolates collected in the Dutch national surveillance. Sci Rep. 2020;10(1):16778.
19. Wielders CCH, Schouls LM, Woudt SHS, Notermans DW, Hendrickx APA, Bakker J, et al. Epidemiology of carbapenem-resistant and carbapenemase-producing Enterobacterales in the Netherlands 2017-2019. Antimicrob Resist Infect Control. 2022;11(1):57.
20. Carattoli A, Zankari E, García-Fernández A, Voldby Larsen M, Lund O, Villa L, et al. In silico detection and typing of plasmids using PlasmidFinder and plasmid multilocus sequence typing. Antimicrob Agents Chemother. 2014;58(7):3895–903.

21. Wick RR, Judd LM, Gorrie CL, Holt KE. Unicycler: Resolving bacterial genome assemblies from short and long sequencing reads. PLoS Comput Biol. 2017;13(6):e1005595.
22. Schwengers O, Jelonek L, Dieckmann MA, Beyvers S, Blom J, Goesmann A. Bakta: rapid and standardized annotation of bacterial genomes via alignment-free sequence identification. Microb Genom. 2021;7(11).
23. Vendrik KEW, de Haan A, Witteveen S, Hendrickx APA, Landman F, Notermans DW, et al. A prospective matched case-control study on the genomic epidemiology of colistin-resistant Enterobacterales from Dutch patients. Commun Med (Lond). 2022;2:55.
24. Robertson, James, and John H E Nash. MOB-suite: software tools for clustering, reconstruction and typing of plasmids from draft assemblies. Microbial genomics vol. 4,8 (2018): e000206.
25. Robertson, James et al. Universal whole-sequence-based plasmid typing and its utility to prediction of host range and epidemiological surveillance. Microbial genomics vol. 6,10 (2020): mgen000435. .
26. Pritchard L, Glover RH, Humphris S, Elphinstoneb JG, Tothc IK. Genomics and taxonomy in diagnostics for food security: soft-rotting enterobacterial plant pathogens. Anal. Methods 8, 12-24
27. National Center for Biotechnology Information. Standard Nucleotide Basic Local Alignment Search Tool. Available from: https://blast.ncbi.nlm.nih.gov/Blast.cgi?PROGRAM=blastn&PAGE_TYPE=BlastSearch&LINK_LOC=blasthome (Accessed on the 23rd of October 2024).
28. Spadar A, Perdigão J, Campino S, Clark TG. Genomic analysis of hypervirulent Klebsiella pneumoniae reveals potential genetic markers for differentiation from classical strains. Sci Rep. 2022;12(1):13671.
29. Chen YT, Chang HY, Lai YC, Pan CC, Tsai SF, Peng HL. Sequencing and analysis of the large virulence plasmid pLVPK of Klebsiella pneumoniae CG43. Gene. 2004;337:189–98.
30. Wu KM, Li LH, Yan JJ, Tsao N, Liao TL, Tsai HC, et al. Genome sequencing and comparative analysis of Klebsiella pneumoniae NTUH-K2044, a strain causing liver abscess and meningitis. J Bacteriol. 2009;191(14):4492–501.
31. Lam MMC, Wyres KL, Duchêne S, Wick RR, Judd LM, Gan YH, et al. Population genomics of hypervirulent Klebsiella pneumoniae clonal-group 23 reveals early emergence and rapid global dissemination. Nat Commun. 2018;9(1):2703.
32. Dong N, Lin D, Zhang R, Chan EW, Chen S. Carriage of blaKPC-2 by a virulence plasmid in hypervirulent Klebsiella pneumoniae. J Antimicrob Chemother. 2018;73(12):3317–21.
33. Xie Y, Tian L, Li G, Qu H, Sun J, Liang W, et al. Emergence of the third-generation cephalosporin-resistant hypervirulent Klebsiella pneumoniae due to the acquisition of a self-transferable bla(DHA-1)-carrying plasmid by an ST23 strain. Virulence. 2018;9(1):838–44.
